# Identification of HIV-1 Vif Regions Required for CBF-β Interaction and APOBEC3 Suppression

**DOI:** 10.1371/journal.pone.0095738

**Published:** 2014-05-08

**Authors:** Hong Wang, Bin Liu, Xin Liu, Zhaolong Li, Xiao-Fang Yu, Wenyan Zhang

**Affiliations:** 1 Institute of Virology and AIDS Research, First Hospital of Jilin University, Changchun, Jilin Province, China; 2 Department of Hand Surgery, First Hospital of Jilin University, Changchun, Jilin Province, China; 3 Department of Molecular Microbiology and Immunology, Johns Hopkins Bloomberg School of Public Health, Baltimore, Maryland, United States of America; University Hospital Zurich, Switzerland

## Abstract

Human immunodeficiency virus type 1 (HIV-1) Vif requires core binding factor β (CBF-β) to degrade the host APOBEC3 restriction factors. Although a minimum domain and certain amino acids of HIV-1 Vif, including hydrophobic residues at the N-terminal, have been identified as critical sites for binding with CBF-β, other regions that potentially mediate this interaction need to be further investigated. Here, we mapped two new regions of HIV-1 Vif that are required for interaction with CBF-β by generating a series of single-site or multiple-site Vif mutants and testing their effect on the suppression of APOBEC3G (A3G) and APOBEC3F (A3F). A number of the mutants, including G84A/SIEW86-89AAAA (84/86–89), E88A/W89A (88/89), G84A, W89A, L106S and I107S in the ^84^GxSIEW^89^ and L^102^ADQLI^107^ regions, affected Vif function by disrupting CBF-β binding. These Vif mutants also had altered interactions with CUL5, since CBF-β is known to facilitate the binding of Vif to CUL5. We further showed that this effect was not due to misfolding or conformational changes in Vif, as the mutants still maintained their interactions with other factors such as ElonginB, A3G and A3F. Notably, G84D and D104A had stronger effects on the Vif-CUL5 interaction than on the Vif-CBF-β interaction, indicating that they mainly influenced the CUL5 interaction and implying that the interaction of Vif with CUL5 contributes to the binding of Vif to CBF-β. These new binding interfaces with CBF-β in HIV-1 Vif provide novel targets for the development of HIV-1 inhibitors.

## Introduction

The Vif protein of human immunodeficiency virus type 1 (HIV-1) is necessary for viral replication and survival in non-permissive cell lines such as H9 and HUT78, which possess an intracellular resistance factor initially designated APOBEC3G (A3G) [4]. Members of the APOBEC3 family of polynucleotide cytidine deaminases were later discovered to have antiviral or anti-retrotransposon activity to different degrees [5], [6]. In order to counteract host restriction factors, HIV-1/SIV Vif proteins all form the E3 ubiquitin ligase by hijacking Cullin5, ElonginB/ElonginC (ELOB/C) to target the cellular antiviral APOBEC3 proteins for degradation [10]–[15]. Core binding factor β (CBF-β), a newly identified Vif regulator, is critical for the Vif-mediated degradation of these APOBEC3 family proteins [1], [7]. Like the Vif protein of HIV-1 subtype B, Vif proteins of many other HIV-1 subtypes and of simian immunodeficiency virus (SIV) also need CBF-β to degrade their respective Vif-sensitive APOBEC3 proteins [8]. CBF-β, a non-DNA binding subunit, heterodimerizes with Runx proteins to form the CBF family of transcription factors, which are important for cell differentiation and proliferation, hematopoiesis and bone development [9], [10]. Recent studies have shown that CBF-β increases the stability of HIV-1 Vif [7], [11], controls its binding to CUL5 by specifically interacting with Vif [1], as well as increases the solubility of Vif when co-expressed *in vitro* [12]. Different domains of CBF-β are required for the Vif-CBF-β and Runx-CBF-β interactions [13]. Difficulties in producing full-length soluble Vif for *in vitro* experimental analyses have been overcome with the discovery of the involvement of CBF-β in Vif function, and the crystal structure of the Vif-CBF-β-CUL5-ELOB/C complex was resolved recently by Guo *et al.* [14].

Previous studies have already identified functional domains of HIV-1 Vif that interact with various cellular factors and with the host restrictive factor APOBEC3G (A3G) or APOBEC3F (A3F). HIV-1 or SIV Vif binds to ElonginB and ElonginC (ELOB/C) through a virus-specific BC box [15],[16]. Primate lentiviral Vif proteins also use a highly conserved H-X_5_-C-X_17–18_C_3–5_-H motif upstream of the BC box and the Cullin5-box to bind CUL5 [17], [18]. The N-terminal domain of Vif also is important for APOBEC3 binding [19], [20]. Although we and other groups have shown that the N-terminal amino acids as well as dispersed and conserved hydrophobic residues in HIV-1 Vif are important for the interaction with CBF-β [1], [2], [3], it is not clear whether other regions of HIV-1 Vif are also required for this interaction.

In the current study, we identified two regions of HIV-1 Vif required for binding to CBF-β. We generated a series of Vif single-site or multiple-site mutants and tested both their interaction with CBF-β and their ability to suppress APOBEC3 function. The results showed that some of the mutations, as a result of their disrupting the interactions with CBF-β and CUL5, affected the ability of Vif to suppress the antiviral activity of A3G. Two exceptions to this pattern were G84D and D104A, which had a stronger effect on the Vif-CUL5 interaction than on the Vif-CBF-β interaction. Thus, we have identified two new functional motifs of Vif that are important for its critical interaction with CBF-β.

## Results

### ^84^GxSIEW^89^ and L^102^ADQLI^107^ in HIV-1 Vif Affect its Interaction with CBF-β and/or CUL5

**Figure 1:**
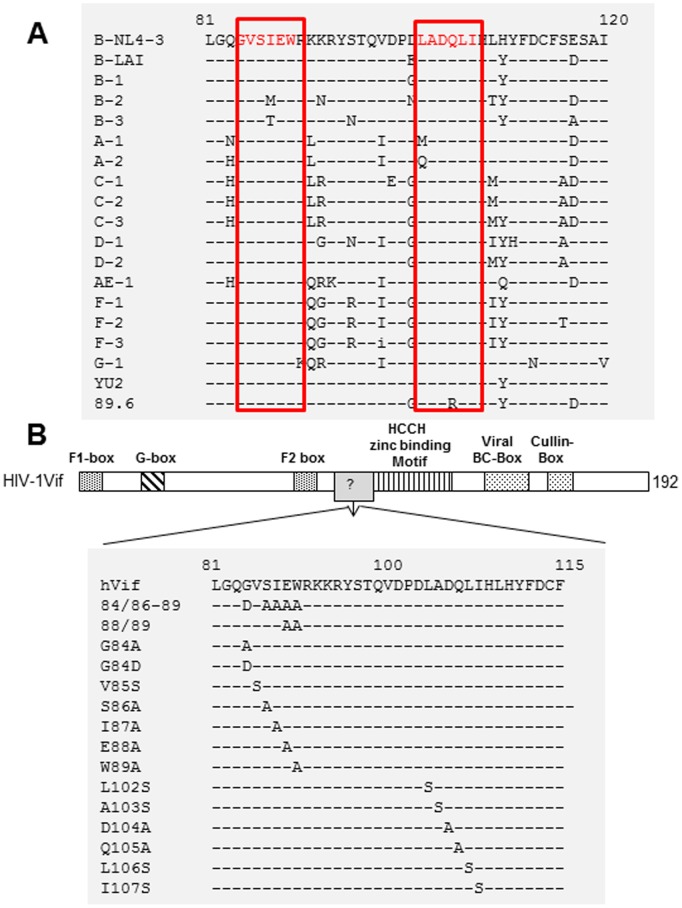
Identification of two highly conserved regions in Vif proteins of various HIV-1 subtypes. (A) Conserved amino acids in Vif molecules. (B) Illustration of Vif mutant constructs.

**Figure 2:**
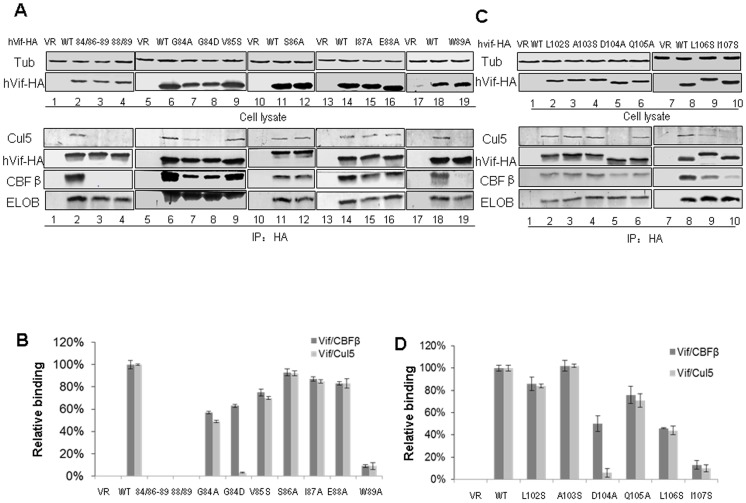
Identification of two regions in HIV-1 Vif that are required for the interaction with CBF-β. (A) Interaction of Vif mutants in the ^84^GxSIEW^89^ region with cellular factors. HEK293T cells were transfected with the vector control, WT or a Vif mutant. Cells were harvested 48 h later and subjected to immunoprecipitation analysis using the anti-HA antibody conjugated to agarose beads. Co-precipitated proteins were analyzed by immunoblotting with antibodies against Vif-HA, CUL5, CBF-β and ELOB. (C) Interaction of Vif mutants in the L^102^ADQLI^107^ region with cellular factors. HEK293T cells were transfected with the vector control, WT or a Vif mutant. Cells were harvested 48 h later and subjected to immunoprecipitation analysis using the anti-HA antibody conjugated to agarose beads. Co-precipitated proteins were analyzed by immunoblotting using antibodies against Vif-HA, CUL5, CBF-β and ELOB. (B and D) Relative binding capacity of WT Vif (100%) and Vif mutants to CBF-β or CUL5. Error bars indicate the standard deviation from triplicate experiments.

Two highly conserved regions, ^84^GxSIEW^89^ and L^102^ADQLI^107^, as indicated by the alignment of Vif proteins from various HIV-1 subtypes (Fig. 1A), have been determined to be critical for APOBEC3 protein suppression [21], [22], [23]. However, the exact functional mechanisms by which these two regions in HIV-1 Vif suppress APOBEC3 proteins are unclear. Therefore, we investigated whether these two regions are important for interactions with other cellular factors, especially the novel regulatory factor CBF-β. Many functional domains of HIV-1 Vif that are involved in interactions with cellular factors and APOBEC3 proteins have been identified (Fig. 1B). We first generated a series of amino acid substitutions in ^84^GxSIEW^89^ and L^102^ADQLI^107^, as indicated in Fig. 1B, and then examined the interactions between these Vif mutants and cellular factors, including ELOB, CUL5 and CBF-β. HEK293T cells were transfected with the negative control vector VR1012, wild-type (WT) Vif or a Vif mutant (Fig. 2A and C). At 48 h after transfection, the cells were harvested, lysed and then loaded onto HA agarose-conjugated beads for immunoprecipitation. The WT Vif could efficiently co-immunoprecipitate ELOB, CBF-β and CUL5 (Fig. 2A, lanes 2, 6, 11, 14 and 18; Fig. 2C, lanes 2 and 8). These factors were not co-immunoprecipitated in the absence of Vif, indicating the specificity of the assay (Fig. 2A, lanes 1, 5, 10, 13 and 17; Fig. 2C, lanes 1 and 7). Several mutants, including 84/86–89, 88/89, G84A and W89A, all interacted with ELOB but were defective in interacting or completely failed to interact with CBF-β and CUL5 (Fig. 2A, lanes 3, 4, 7 and 19). We also found that the L106S and I107S mutants (Fig. 2C, lanes 9 and 10) showed a defective interaction or completely failed to interact with CBF-β and CUL5, as compared to WT Vif and the mutants L102S and A103S (Fig. 2C, lanes 2, 3 and 4). It is worth noting that G84D and D104A totally lost the ability to interact with CUL5 and showed a reduced level of interaction with CBF-β (Fig. 2A, lane 8 and Fig. 2C, lane 5). In repeated experiments, 84/86–89, 88/89, G84A, W89A, L106S and I107S showed a 40–90% reduction in CBF-β and CUL5 binding when compared with WT Vif (Fig. 2B and D). Consistent with a previous determination that shutting down CBF-β affects the binding of Vif to CUL5 [1], we deduced that the above mutants had lost the ability to interact with CBF-β and therefore could not interact with CUL5. It is possible that G84D and D104A affected the binding of Vif to CUL5 but not to CBF-β. These results indicated that key amino acids in these two regions of HIV-1 Vif are indeed required for the interaction between Vif and CBF-β.

### ^84^GxSIEW^89^ and L^102^ADQLI^107^ in HIV-1 Vif are Defective in Inhibiting the Antiviral Activity of A3G

**Figure 3:**
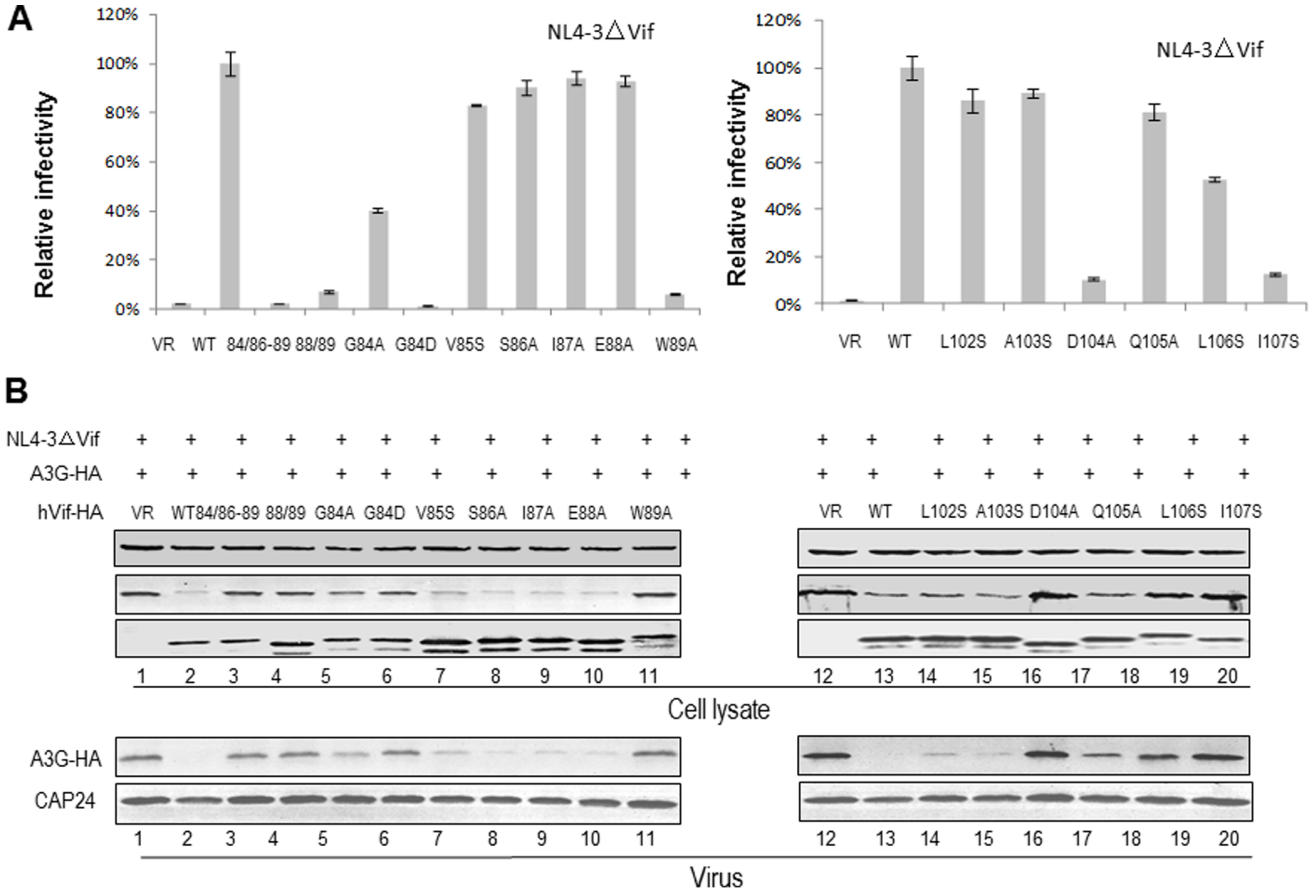
Effect of Vif mutants on the antiviral activity of A3G. HEK293T cells were transfected with NL4-3ΔVif plus A3G in the presence of WT or mutant Vif as indicated. After 48 h, HIV-1 viruses were harvested. (A) Virus infectivity was assessed using MAGI indicator cells, with the virus infectivity in the presence of WT Vif set to 100%. Error bars indicate the standard deviation from triplicate experiments. (B) A3G stability was assessed by immunoblotting against A3G-HA, Vif-HA and tubulin as a loading control. A3G packaging was evaluated by immunoblotting against A3G-HA and CAp24 after viruses were isolated by ultracentrifugation from the supernatants of cell cultures.

Since some amino acids in ^84^GxSIEW^89^ and L^102^ADQLI^107^ of HIV-1 Vif were found to affect its interaction with CBF-β and/or CUL5, we examined whether these amino acids could inhibit antiviral activity of A3G. HEK293T cells were transfected with the infectious molecular clone of the Vif mutant, NL4-3ΔVif and A3G expression vector plus VR1012 (as a control vector), WT Vif or a Vif mutant. Virus was produced from transfected cells and tested for infectivity in a standard multinuclear activation of galactosidase indicator (MAGI) assay. WT Vif could suppress A3G activity and maintained NL4-3ΔVif infectivity, and this level was considered to be 100% for assessing relative infectivity with the Vif mutants (Fig. 3). As expected, A3G dramatically reduced the infectivity of NL4-3ΔVif in the absence of Vif. The 84/86–89, 88/89, G84D, G84A and W89A mutants in the ^84^GxSIEW^89^ region showed a reduced ability to suppress the antiviral activity of A3G, resulting in lowered virus infectivity (Fig. 3A). The D104A, L106S and I107S mutations in the L^102^ADQLI^107^ region also reduced the ability of Vif to suppress the antiviral activity of A3G to different extents (Fig. 3B). In agreement with the binding data, V85S and Q105A had only a small effect on Vif function.

A3G protein expression was also detected in viral producer cells by immunoblotting. We found that the 84/86–89, 88/89, G84D, G84A, W89A, D104A, L106S and I107S mutants all failed to decrease A3G expression. Consistent with the results obtained from the viral infectivity assays, the results show that these Vif mutants, which have either lost or have a deficit in the ability to interact with CBF-β and/or CUL5, also have a decreased ability to inhibit A3G.

### HIV-1 Vif Proteins with Mutations in Certain Amino Acids in the ^84^GxSIEW^89^ and L^102^ADQLI^107^ Regions are Defective in Degrading A3G and Inhibiting its Incorporation into Virions

**Figure 4:**
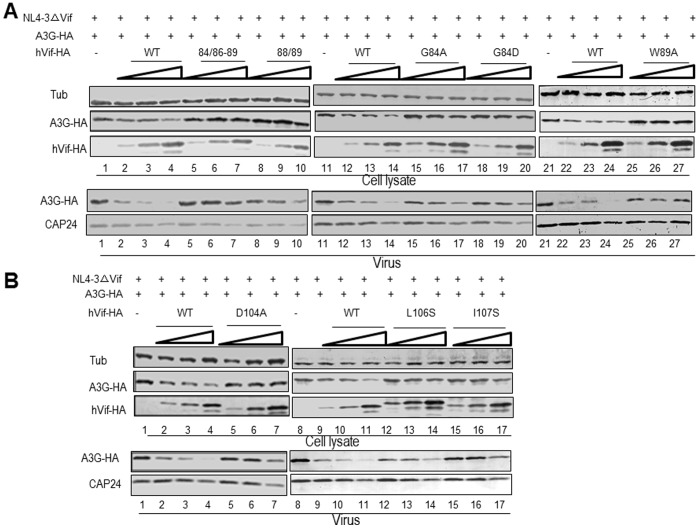
Effect of Vif mutants on A3G degradation and virion packaging. HEK293T cells were transfected with 0.5 µg NL4-3ΔVif plus 0.5 µg A3G in the presence of increasing doses of WT Vif or mutants as indicated. A3G stability was assessed by immunoblotting against A3G-HA, Vif-HA and tubulin as a loading control. A3G packaging was evaluated by immunoblotting against A3G-HA and CAp24 after viruses were isolated by ultracentrifugation from the supernatants of cell cultures.

In order to further investigate whether differences of Vif sensitivity to A3G are caused by the different doses or mutations of Vif, we transfected HEK293T cells with NL4-3ΔVif and A3G expression vector plus either VR1012 (as a control vector), a 2-fold higher amount of WT, or the 84/86–89, 88/89, G84D, G84A, W89A, D104A, L106S or I107S construct. In agreement with results of the infectivity assay, the intracellular level of A3G was gradually reduced when the amount of WT Vif was increased (Fig. 4A, lanes 2–4, 12–14 and 22–24; Fig. 4B, lanes 2–4 and 9–11), when compared to control VR1012 (Fig. 4A, lanes 1, 11 and 21; 4B, lanes 1 and 8). Mutants 84/86–89 (Fig. 4A, lanes 5–7), 88/89 (Fig. 4A, lanes 8–10), G84A (Fig. 4A, lanes 15–17), G84D (Fig. 4A, lanes 18–20), W89A (Fig. 4A, lanes 25–27), D104 (Fig. 4B, lanes 5–7), L106S (Fig. 4B, lanes 12–14) and I107S (Fig. 4B, lanes 15–17) were unable to efficiently degrade A3G, even at high expression levels. Consequently, these Vif mutants also could not efficiently inhibit the incorporation of A3G into virions, when compared to WT Vif (Fig. 4).

### Mutations in HIV-1 Vif Disrupting the Interaction with CBF-β and/or CUL5 do not Influence its Interaction with A3G or A3F

**Figure 5:**
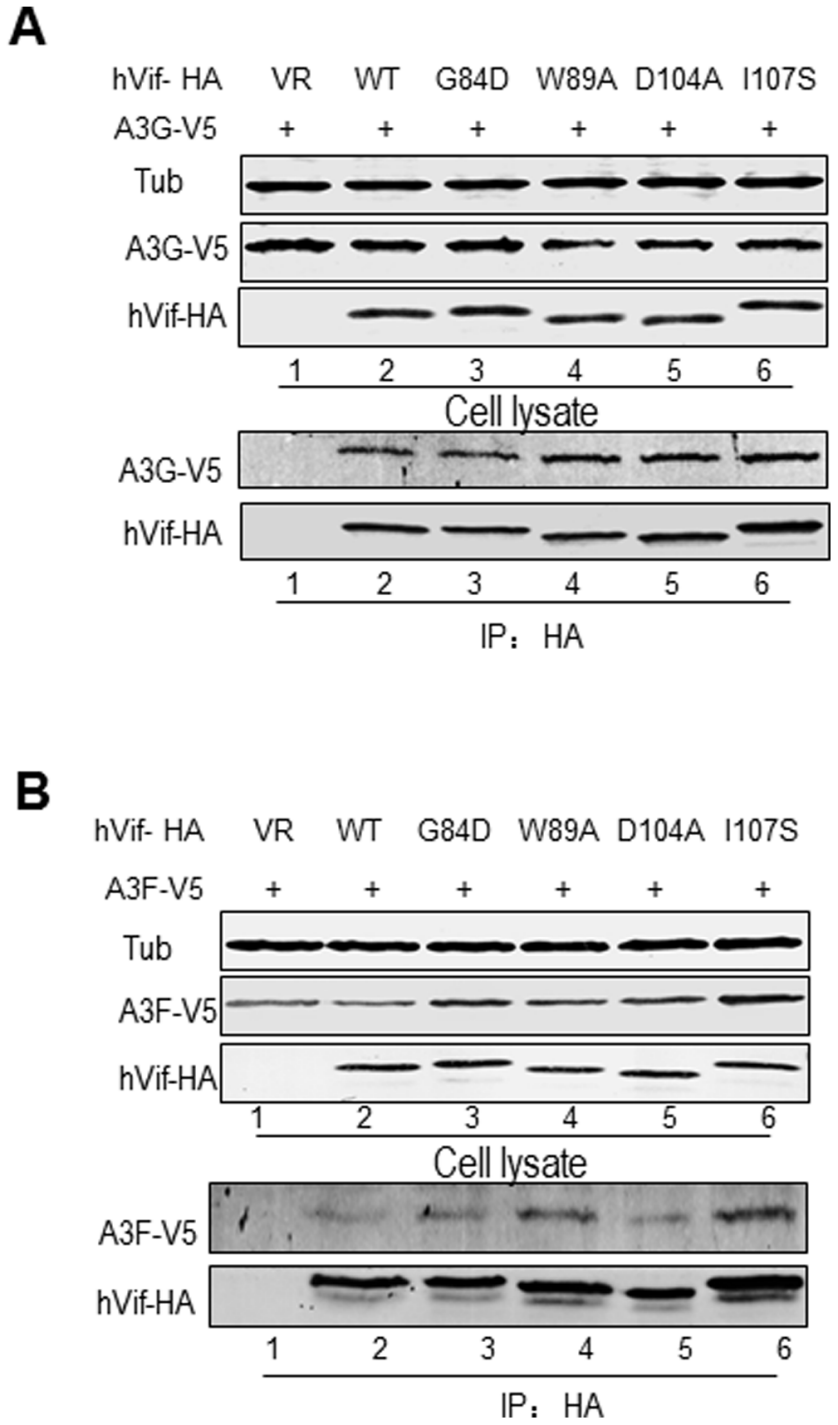
Vif mutants maintain interactions with A3G and A3F. HEK293T cells were transfected with the vector control, WT or a Vif mutant plus A3G-V5 (A) or A3F-V5 (B). Cells were treated with 10 µM MG132 12 h prior to harvesting, and then Vif-HA was immunoprecipitated from cell lysates with an anti-HA antibody conjugated to agarose beads. The interaction of Vif-HA with A3G-V5 or A3F-V5 was detected by immunoblotting using antibodies against Vif-HA and A3G-V5 or A3F-V5.

As we had shown that Vif mutants of critical residues that were defective for binding to CBF-β and/or CUL5 could still bind to ELOB, we wanted to determine whether these mutations induce a conformational change in Vif by examining their ability to bind to the target protein A3G and another cytidine deaminase, A3F. Therefore, we transfected HEK293T cells with the negative control vector VR1012, WT Vif, G84D, W89A, D104A or I107S mutant plus A3G-V5 or A3F-V5. The cells were treated with 10 µM MG132 for 12 h prior to harvesting. At 48 h after transfection, the cells were harvested, lysed and then loaded onto HA agarose-conjugated beads for immunoprecipitation. All of the Vif mutants could efficiently co-immunoprecipitate A3G and A3F, when compared to WT Vif (Fig. 5A and 5B, lane 2). This interaction was specific since A3G-V5 and A3F-V5 were not detected in the absence of Vif (Fig. 5A and 5B, lane 1). These results showed that the mutants were not misfolded and that they had not induced conformational changes that abolished the interaction of Vif with CBF-β and/or CUL5.

### Mutations in HIV-1 Vif Disrupting the Interaction with CBF-β and/or CUL5 Affect A3F Expression

**Figure 6:**
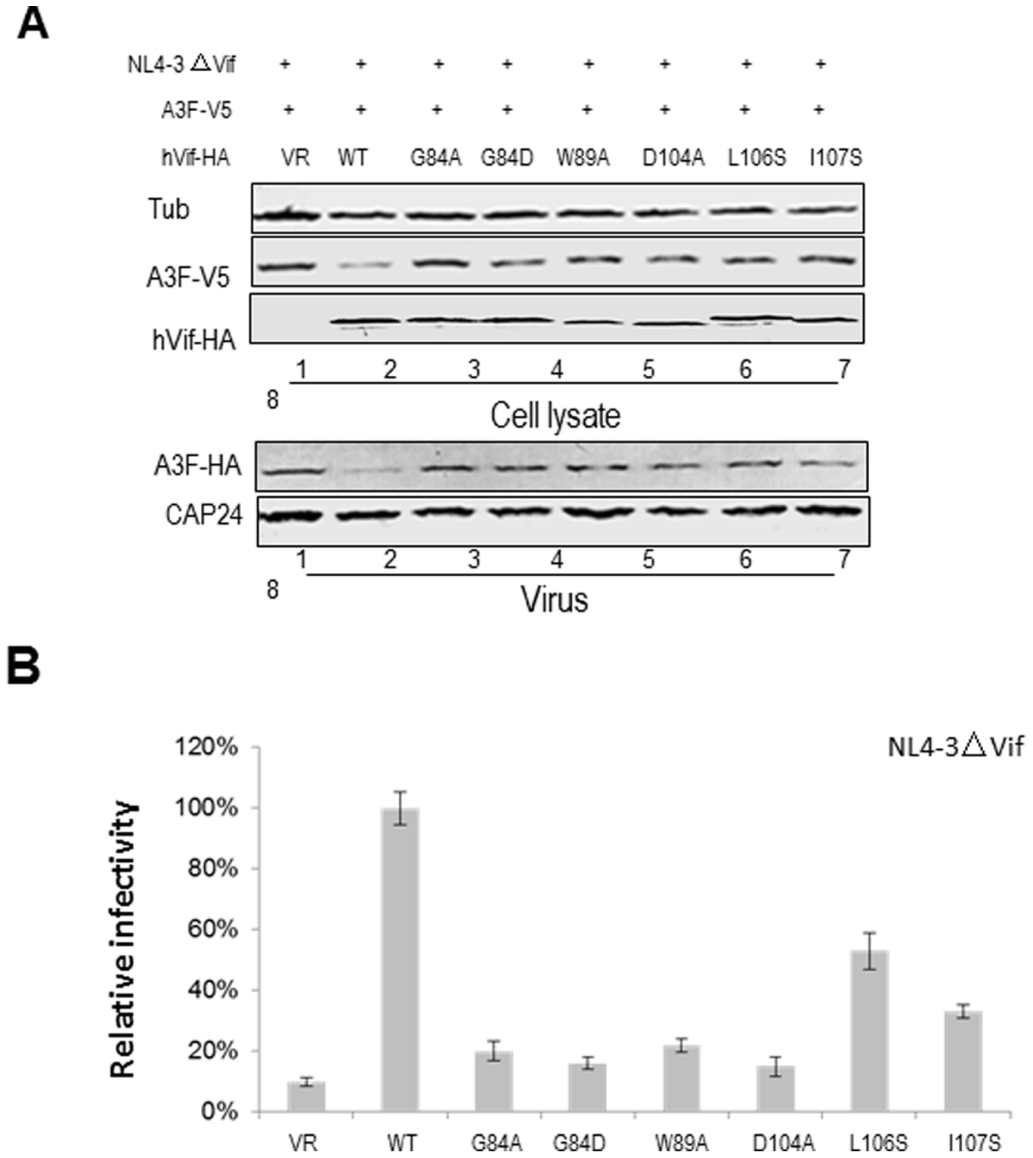
HIV-1 Vif proteins with mutations that abolish the interaction with CBF-β and/or CUL5 are defective in degrading A3F. HEK293T cells were transfected with the control vector, WT Vif or a Vif mutant as indicated and harvested after 48-HA, tubulin and A3F-V5. Error bars indicate the standard deviation from triplicate experiments.

We also examined whether the important Vif mutants G84A, G84D, 84/86–89, 88/89, W89A, D104, L106S and I107S, which all affected the interaction with CBF-β and/or CUL5, could also affect the expression of A3F. HEK293T cells were transfected with A3F plus the negative control vector VR1012, WT Vif or a Vif mutant. As expected, we observed that the intracellular level of A3F was reduced to a greater extent by WT Vif (Fig. 6A, lane 2) than by the VR1012 control (Fig. 6A, lane 1). Vif mutants G84A, G84D, W89A, 84/86–89, 88/89, D104, L106S and I107S resulted in increased A3F expression and showed a reduced ability to neutralize the antiviral activity of A3F when compared to WT Vif (Fig. 6B). These results showed that mutants that had defects in the interaction with CBF-β and/or CUL5 also had a compromised ability to degrade and inhibit A3F.

## Discussion

In the current study, we have for the first time identified two regions of HIV-1 Vif that affect the Vif-mediated degradation of A3G and A3F by disrupting its interaction with CBF-β and/or CUL5. Except for G84D and D104A, the mutations studied, including 84/86–89, 88/89, G84A, W89A, L106S and I107S, in the ^84^GxSIEW^89^ and L^102^ADQLI^107^ regions of HIV-1 Vif played important roles in the Vif-CBF-β interaction. Our study is consistent with the recent report published by Zhou *et al.* indicating the involvement of a minimum fragment (5–126) in HIV-1 Vif with CBF-β binding [3].

Analysis of alanine or serine substitutions in HIV-1 Vif showed that G84A, 84/86–89, 88/89, W89A, L106S and I107S (Fig. 1) caused poor interaction with CBF-β, especially W89A and I107S. V85S and Q105A only slightly affected the interaction of Vif with CBF-β. Hultquist *et al.* reported that Vif proteins of various HIV-1 subtypes and SIV all require CBF-β for the degradation of A3G [8]. We observed that the amino acids mentioned above are all highly conserved in Vif proteins of various HIV-1 subtypes, as indicated by alignment analysis (Fig. 1A). Taking into consideration the involvement of W21 and W38 in this interaction as previously shown [1], we and other groups have further indicated that Vif simultaneously uses an extended interface encompassing several domains to bind CBF-β [2] [3]. In our study, it was noteworthy that G84D and D104 totally abolished Cul5 binding, but these mutants still retained some interaction with CBF-β. Therefore, it is possible that G84D and D104 directly affect the interaction of Vif with CUL5, which contributes to or stabilizes the Vif-CBF-β interaction. Fribourgh *et al.* recently also proposed that CUL5 binding enhances the stability of the Vif-CBF-β interaction [24]. Consistent with the above findings, functional experiments further confirmed that Vif mutations at these amino acids affected the interaction with CBF-β and/or CUL5 as well as destroyed the ability to inhibit antiviral activity of A3G and A3F (Fig. 3 and 6).

Although the Vif mutants listed above could not interact with CBF-β and/or CUL5, we could not exclude the possibility that they were misfolded non-functional proteins or had induced conformational changes that eliminated those interactions. Investigation of the interaction between Vif mutants and other cellular factors, ELOB, A3G and A3F showed that they did not undergo conformational changes and indeed specifically failed to interact with CBF-β and/or CUL5 (Figs. 2 and 5).

**Figure 7:**
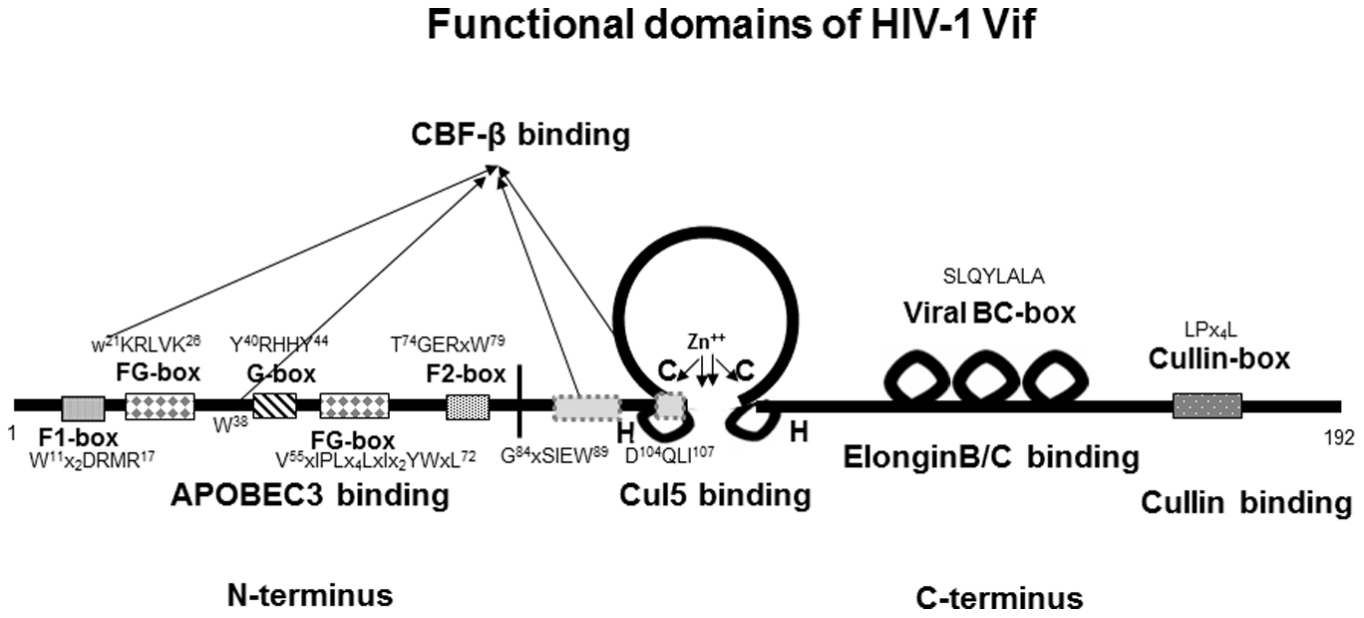
Schematic of HIV-1 Vif functional domains. The α-helix of the BC-box mediates the interaction with ElonginC. A zinc-binding H-X_5_-C-X_17–18_C_3–5_-H motif and Cullin-box are important for the CUL5 interaction. Vif mutants of W21, W38 and the newly identified discontinuous domains (including G^84^xSIEW^89^ and L^102^ADQLI^107^) are defective in binding CBF-β and/or CUL5. The N-terminus of Vif mediates the interaction with APOBEC3 proteins.

As a substrate receptor, Vif molecules not only recruit ELOB/C, Rbx1 and CBF-β to form the E3 ubiqutin ligase but also need to recognize different APOBEC3 proteins for degradation. The various functional domains of Vif have been well analyzed (Fig. 7). In HIV-1 Vif, the Y^40^RHHY^44^ motif is only involved in A3G binding [25], while D^14^RMR^17^ and T^74^GERxW^79^ are both involved in A3F binding [19], [25]. The binding sites for both A3G and A3F are W^21^KSLVK^26^, V^55^xIPLx_4–5_LxΦYWxL^72^ and Y^69^xxL^72^ [19], [26], [27], [28]. Except in the case of the W21 and W38 sites, discontinuous G^84^xSIEW^89^ and L^102^ADQLI^107^ domains interact with CBF-β. Therefore, it would be interesting to distinguish the exact mechanism by which Vif interacts with ELOB/C, CUL5, CBF-β and the target APOBEC3 proteins. Crystal structural information on the Vif-CBF-β-CUL5-ELOB/C complex recently revealed by Guo *et al.* has provided valuable insight into the exact binding sites and mechanism of interaction, which is very helpful for understanding the biochemical basis of Vif-host interactions [14]. Building on the accumulated evidence discussed above, the discovery in this study of new HIV-1 Vif domains capable of binding CBF-β may lead to the development of novel therapeutic strategies for HIV-1 infection.

## Materials and Methods

### Plasmid Construction

The infectious molecular clone of the Vif mutant pNL4-3ΔVif construct was obtained from the AIDS Reagents Program, Division of AIDS, National Institute of Allergy and Infectious Diseases (NIAID), National Institutes of Health (NIH). The sequences coding for the full-length Vif protein (phVif) from NL4-3 (residues 1–192) were codon-optimized, constructed with an C-terminal HA tag and inserted between the *Eco*RI and *Bam*HI sites of VR1012. Plasmids phVif G84A, G84D, V85S, S86A, I87A, E88A, W89A, G84A/SIEW86–89AAAA(84/86–89), E88A/W89A(88/89), L102A, A103S, D104A, Q105A, L106A and I107A were derived from phVif-HA by site-directed mutagenesis. The V5-tagged human A3F-expressing vector pcDNA3-A3F-V5 was a kind gift of YH Zheng and BM Peterlin at University of California, San Francisco [29]. The expression vectors pc-hA3G-HA and pc-hA3G-V5 were described previously [30].

### Cell Culture, Transfection and Antibodies

MAGI-CCR5 was obtained through the NIH AIDS Research Reagent Program (cat. no. 3522) from Julie Overbaugh [31]. The human HEK293T (ATCC, cat. no. CRL-11268) and MAGI-CCR5 cells were maintained in Dulbecco’s modified Eagle’s medium (DMEM, Invitrogen) with 10% fetal bovine serum and penicillin/streptomycin (D-10 medium) and passaged upon confluency. DNA transfection was carried out using polyethylenimine (PEI) reagent according to the manufacturer’s instructions (Polyscience, cat. no. 23966-2). The following antibodies or sera were used in this study: β-tubulin monoclonal antibody (mAb; Covance, cat. no. NMS-410P), anti-CUL5 (H-300) rabbit polyclonal antibody (Santa Cruz Biotechnology, cat. no. sc-13014), anti-ElonginB (FL-118) rabbit polyclonal antibody (Santa Cruz Biotechnology, cat. no. sc-1144), anti-V5 mAb (Invitrogen, cat. no. R960-25), anti-CBFβ mAb (Santa Cruz Biotechnology, cat. no. sc-166142), CAp24 mAb (NIH AIDS Reagents Program, cat no. 1513) and anti-HA rabbit polyclonal antibody (Santa Cruz Biotechnology, cat. no. 71–5500).

### Virus Purification and Viral Infectivity (MAGI) and A3G Degradation Assays

For A3G degradation and viral infectivity assays, HEK293T cells were transfected with 0.5 µg NL4-3ΔVif plus 0.5 µg A3G-HA in the presence of increasing doses of WT or mutant Vif in six-well plates. Viruses produced by transfected HEK293T cells were harvested from cell culture supernatants, and cells were reserved for detection of A3G degradation. Virus in cell culture supernatants was cleared of cellular debris by centrifugation at 3,000 rpm for 15 min in an Eppendorf centrifuge and filtration through a 0.22-µm pore size membrane (Millipore). Virus particles were then concentrated by ultracentrifugation through a 20% sucrose cushion at 100,000×*g* for 2 h at 4°C in a Beckman Optial-100XP ultracentrifuge. Viral pellets were resuspended in lysis buffer (PBS containing 1% Triton X-100 and complete protease inhibitor cocktail [Roche]). Viral lysates were analyzed by immunoblotting.

Viral infection was determined by the MAGI assay [31] as follows. MAGI-CCR5 cells were prepared in 24-well plates in D-10 medium 1 day before infection. On the day of infection, cells at 30–40% confluency were infected by removing the medium from each well and adding dilutions of virus in a total volume of 500 µl of complete DMEM with 20 µg of DEAE-dextran per well. After a 2-h incubation at 37°C in a 5% CO_2_ incubator, 500 µl of complete DMEM was added to each well, and the cells were incubated for 48 h under the same conditions. The supernatants were removed, and 500 µl of fixing solution (1% formaldehyde, 0.2% glutaraldehyde in PBS) was added. After a 5-min incubation, the cells were washed twice with PBS. The staining solution (20 µl of 0.2 M potassium ferrocyanide, 20 µl of 0.2 M potassium ferricyanide, 2 µl of 1 M MgCl_2_ and 10 µl of 40 mg/ml 5-bromo-4-chloro-3-indolyl-β-D-galactopyranoside [X-Gal]) was added. Cells were incubated for 2 h at 37°C in a non-CO_2_ incubator. Staining was stopped by removing the staining solution, and the cells were thoroughly washed twice with PBS. In this system, β-galatosidase activity is under the control of the HIV-1 LTR promoter, which is trans-activated in the presence of integrated virus. Positive blue dots produced after addition of X-gal substrate were counted, and viral infectivity was determined after normalizing the amount of input virus in terms of the p24 antigen content. All infectivity assays were performed in triplicate.

### Immunoprecipitation and Immunoblot Analysis

To determine whether these Vif mutants can bind to CBF-β and other factors in the CUL5 E3 ligase complex, HEK293T cells were transfected with 1 µg of WT or mutant Vif expression vector in six-well plates. To assess the interaction between Vif and A3G or A3F, the ratio of Vif to A3G or A3F was 1∶3 (0.5 µg and 1.5 µg). MG132 (Sigma, cat. no. C2211) at the final concentration of 10 µM was added to the cells 12 h before harvest. Cells were harvested, washed twice with cold PBS and resuspended in lysis buffer (50 mM Tris-HCl [pH 7.5], 150 mM NaCl, 0.5% [v/v] NP-40 and complete protease inhibitor cocktail tablets) at 4°C for 30 min, followed by centrifugation at 13,000 rpm for 30 min. MG132 was dissolved in DMSO and made at the stock concentration of 10 mM. For HA-tag immunoprecipitation, pre-cleared cell lysates were mixed with anti-HA antibody-conjugated agarose beads (Roche) and incubated at 4°C for 3 h. Samples were then washed six times with washing buffer (20 mM Tris-HCl [pH 7.5], with 100 mM NaCl, 0.1 mM EDTA and 0.05% [v/v] Tween-20). The beads were eluted with elution buffer (0.1 mM glycine-HCl, pH 2.0) or 4× loading buffer. The eluted materials were subsequently analyzed by immunoblotting.

### Sequence Alignment of Vif from various HIV-1 Subtypes

Sequences of Vif proteins from HIV-1 subtypes A, B, C, D, AE, F and G were obtained from Viviana Simon [8]. NCBI reference sequence numbers for the subtypes YU2 and 89.6 used are M93258.1 and U39362.2, respectively.
